# Cognitive and affective theory of mind in dementia with Lewy bodies and Alzheimer’s disease

**DOI:** 10.1186/s13195-016-0179-9

**Published:** 2016-03-16

**Authors:** Camille Heitz, Vincent Noblet, Clélie Phillipps, Benjamin Cretin, Natacha Vogt, Nathalie Philippi, Jennifer Kemp, Xavier de Petigny, Mathias Bilger, Catherine Demuynck, Catherine Martin-Hunyadi, Jean-Paul Armspach, Frédéric Blanc

**Affiliations:** Neuropsychology Unit, Memory Resource and Research Centre (CMRR), Department of Neurology, University Hospital of Strasbourg, Strasbourg, France; ICube Laboratory, IMIS Team, University of Strasbourg, CNRS, FMTS, Strasbourg, France; Day Hospital, Memory Resources and Research Centre (CMRR), Department of Geriatrics, University Hospital of Strasbourg, Strasbourg, France

**Keywords:** Theory of mind, Dementia with Lewy bodies, Alzheimer’s disease, Brain volume, Neural correlates

## Abstract

**Background:**

*Theory of mind* (ToM) refers to the ability to attribute mental states, thoughts (cognitive component) or feelings (affective component) to others. This function has been studied in many neurodegenerative diseases; however, to our knowledge, no studies investigating ToM in dementia with Lewy bodies (DLB) have been published. The aim of our study was to assess ToM in patients with DLB and to search for neural correlates of potential deficits.

**Methods:**

Thirty-three patients with DLB (DLB group) and 15 patients with Alzheimer’s disease (AD group), all in the early stage of the disease, as well as 16 healthy elderly control subjects (HC group), were included in the study. After a global cognitive assessment, we used the Faux Pas Recognition (FPR) test, the Reading the Mind in the Eyes (RME) test and Ekman’s Facial Emotion Recognition test to assess cognitive and affective components of ToM. Patients underwent cerebral 3-T magnetic resonance imaging, and atrophy of grey matter was analysed using voxel-based morphometry. We performed a one-sample *t* test to investigate the correlation between each ToM score and grey matter volume and a two-sample *t* test to compare patients with DLB impaired with those non-impaired for each test.

**Results:**

The DLB group performed significantly worse than the HC group on the FPR test (*P* = 0.033) and the RME test (*P* = 0.015). There was no significant difference between the AD group and the HC group or between the DLB group and the AD group. Some brain regions were associated with ToM impairments. The prefrontal cortex, with the inferior frontal cortex and the orbitofrontal cortex, was the main region, but we also found correlations with the temporoparietal junction, the precuneus, the fusiform gyrus and the insula.

**Conclusions:**

This study is the first one to show early impairments of ToM in DLB. The two cognitive and affective components both appear to be affected in this disease. Among patients with ToM difficulties, we found atrophy in brain regions classically involved in ToM, which reinforces the neuronal network of ToM. Further studies are now needed to better understand the neural basis of such impairment.

**Electronic supplementary material:**

The online version of this article (doi:10.1186/s13195-016-0179-9) contains supplementary material, which is available to authorized users.

## Background

*Theory of mind* (ToM) refers to the capacity to understand someone else’s emotions and mental state. ToM can be separated into two systems: the affective and cognitive components. The affective ToM is involved in drawing inferences about other people’s emotions and feelings; the cognitive ToM is involved in drawing inferences about other people’s beliefs and intentions [1]. Researchers have developed experimental tests to assess this capacity. The main tests used in the literature are the first- and second-order false belief tasks, the Faux Pas Recognition (FPR) test, the Reading the Mind in the Eyes (RME) test and Ekman’s Facial Emotion Recognition test [2]. Neuroimaging and lesion studies have tried to establish the neural correlates of ToM. In their review of the literature, Carrington and Bailey [3] found 40 functional imaging studies about ToM. Prefrontal regions, especially the medial prefrontal and orbitofrontal cortices, were associated with ToM in 93 % of studies. The anterior cingulate cortex, superior temporal sulcus (STS) and temporoparietal junction (TPJ) were involved in 50–60 % of studies. Poletti et al. [4] proposed a model for ToM processing with common brain areas (precuneus, TPJ and STS) for both the affective and cognitive components of ToM, as well as for specific areas for each component. Indeed, the ventromedial prefrontal region would be preferentially involved in affective ToM [5], while cognitive ToM would be supported by the dorsolateral prefrontal cortex [6].

The first publications about ToM reported studies on patients with autism [7] or schizophrenia [8]. In the 2000s, researchers began to assess ToM in neurological diseases. One of the first of these studies was by Gregory et al. [9], who showed ToM impairments in patients with frontotemporal lobe degeneration. These results were confirmed in other studies (for review, see [10]) that revealed a substantial, early and global deficit of both components of ToM in this disease. However, this is not the case in other neurodegenerative diseases, such as Alzheimer’s disease (AD) and Parkinson’s disease (PD). In AD, patients are relatively preserved from ToM impairment at the beginning of the disease, with subtle difficulties in high-demand cognitive tests [9, 11, 12]. In PD, it has been reported that patients develop ToM impairments as the disease progresses, particularly involving the cognitive component [13–15]. Studies on ToM have been carried out in many other neurological diseases, including Huntington’s disease [16], amyotrophic lateral sclerosis [17], corticobasal degeneration [18], semantic dementia [19], progressive supranuclear palsy [20] and multiple sclerosis [21], but no studies have assessed ToM in dementia with Lewy bodies (DLB). DLB is the second most common cause of neurodegenerative dementia after AD in people aged over 65 years. DLB is characterized by intra-neuronal inclusions of α-synuclein, called *Lewy bodies*. In series of demented patients, 15–25 % of patients had pathological lesions of DLB [22]. Patients with DLB sometimes have social behavioural disorders, even in the first stage of the disease [23]. We hypothesised that these disorders are caused by ToM impairments. The only publication evoking ToM in DLB is Modinos et al.’s study [24]. They assessed ToM in five patients with different types of dementia: mild cognitive impairment, AD, frontotemporal dementia, vascular dementia and DLB. Their patient with DLB performed worse than the patient with AD and the one with mild cognitive impairment but better than the patient with frontotemporal dementia. This result is in line with our hypothesis, but a prospective study was needed to confirm this case report.

The aim of our study was to assess affective and cognitive ToM in patients with DLB in the early stage of the disease, and to search for the neural correlates of these deficits. We hypothesised first that patients with DLB would have cognitive and affective ToM impairments and second that any such cognitive deficit would be correlated predominantly to atrophy in the prefrontal cortex and the TPJ.

## Methods

### Selection of participants

We conducted a prospective, single-centre, multi-site study between June 2012 and January 2015 with patients with probable DLB (DLB group) or probable AD (AD group), in all cases in the early stage of the disease, and with healthy elderly control subjects (HC group). Patients were included by six neurologists and geriatricians, experts in dementia, at the Memory Resource and Research Centre of the University Hospital of Strasbourg in France. Healthy elderly subjects were recruited by means of an advertisement for volunteers in a local newspaper. The study was approved by the local ethics committee (Comité de Protection des Personnes Est IV, Strasbourg, France), and all participants gave their written informed consent.

Inclusion criteria for patients with DLB and patients with AD were as follows: age 45 years or older, presence of probable DLB as defined by McKeith’s criteria revised in 2005 [25] and the *Diagnostic and Statistical Manual of Mental Disorders, Fifth Edition* [26], or probable AD as defined by Dubois’s criteria [27] in the early stage of the disease, as defined in both cases by impairment according to results of at least two neuropsychological tests and a Mini Mental State Examination (MMSE) score higher than 20. Patients were excluded in the case of diagnostic doubt, association of AD and DLB, other central neurological or psychiatric disease, visual or hearing disorders or difficulty in speaking French.

Inclusion criteria for healthy subjects were age between 55 and 90 years, absence of neurological or psychiatric history, a normal neurological examination and absence of impairment on neuropsychological tests (excluding ToM tests).

First, the three groups were examined by clinicians with expertise in dementia in order to perform a complete anamnesis and medical examination. Using the Unified Parkinson’s Disease Rating Scale III score [28], akinesia, rigidity and tremor at rest were rated from 0 (no symptoms) to 4 (serious impairment). Fluctuations were assessed with the Mayo Clinic Fluctuation Scale [29] and the Clinician Assessment of Fluctuation [30]. The medical check-up was completed with cerebral magnetic resonance imaging (MRI) for the three groups and lumbar puncture with AD biomarkers (tau, phosphorylated tau and amyloid-β_42_) for the DLB and AD groups. DaTscan (GE Healthcare/Medi-Physics, Arlington Heights, IL, USA), a radiopharmaceutical agent used in single-photon emission computed tomography (SPECT) for striatal dopamine transporter visualization of the brain, was employed if the extrapyramidal syndrome was unclear.

Second, cognitive functions were evaluated using the following tests: MMSE [31] for general cognitive function; the French version of the Free and Cued Selective Reminding Test (FCSRT) [32, 33], the 48-item Delayed Matching to Sample (DMS48) test [34] and the digit span test for memory function; the 80-item Dénomination Orale (DO80; an oral naming test) [35] for language; the Frontal Assessment Battery (FAB) [36], the Trail Making Test (TMT) A and B [37], the Digit Symbol Substitution Test (DSST) [38] and ‘formal and semantic lexical evocation’ [39] for executive function; the praxis set of Mahieux [40] and the Rey-Osterrieth complex figure test [41] for praxis; and number localisation and cube analysis of the Visual Object and Space Perception (VOSP) battery [42] for visuoperceptive functions.

### ToM tasks

ToM was assessed by have participants perform three tasks: the FPR test, the RME test and Ekman’s Facial Emotion Recognition test.

The FPR test [43] is frequently used to assess cognitive and affective components of ToM. We used the shortened version, in which ten stories are read to participants: five containing a social faux pas (faux pas stories) and five control stories without a social faux pas (control stories). For each of the faux pas stories, participants were asked the following questions (faux pas questions): (1) ‘Did anyone say something he or she shouldn’t have said’? (2) ‘Who said something he or she shouldn’t have said’? (3) ‘Why shouldn’t he or she have said it’? (4) ‘Why do you think he or she said it’? (5) ‘Did X know that Y…’? (6) ‘How did X feel’? One point was given for each faux pas question (i.e., a total possible score of 30 points for the 5 faux pas stories). Two points were given for correct rejection of control stories (i.e., a total possible score of ten points for the five control stories). For each story (faux pas and control stories), two control questions were asked to check that participants understood the details of the story. One point was given for each control question (i.e., a total possible score of 20 points for the 10 stories). In our results, the scores for the following questions were also considered separately: questions 3 and 4, assessing the participant’s comprehension of the faux pas; question 5, assessing the participant’s comprehension of the character’s intention; and question 6, assessing the participant’s capacity for attributing emotions to the victim of the faux pas. This last question is used to evaluate affective ToM, and the other questions are used to evaluate cognitive ToM.

The French version of the RME test [44] is used to assess affective ToM on the basis of comprehension of complex mental states. Thirty-six photographs of the eye region of the faces of actors and actresses were presented to participants. Participants were required to choose which of four adjectives best described the mental state. A glossary with all of the proposed adjectives could be used during the assessment.

Ekman’s Facial Emotion Recognition test [45] is also used to assess affective ToM, but with a lower level of difficulty than the RME test. Thirty-five photographs of faces were presented to participants. The participants were asked to choose which of the following emotions corresponded to the facial expression: ‘happiness’ , ‘fear’ , ‘anger’ , ‘disgust’ , ‘surprise’ , ‘sadness’ or ‘neutral’.

Finally, we calculated the global score for the mini-Social cognition and Emotional Assessment (mini-SEA) battery [46] using the FPR test score and Ekman’s Facial Emotion Recognition test score.

For each test, we compared the patients’ results with standardized scores and classified them into successful or failed groups. We used the standardized scores of the mini-SEA [46] for the FPR test and Ekman’s Facial Emotion Recognition test, and Baron-Cohen’s standardised scores [44] were used for the RME test. The score was considered pathological for a *z*-score less than −1.6.

### Scanning protocol

Twenty-four patients with DLB, nine patients with AD and fifteen healthy control subjects underwent cerebral MRI in a 3-T MAGNETOM Verio scanner (Siemens Healthcare, Erlangen, Germany). T1-weighted spin echo sequences were collected using the following parameters: repetition time = 1900 ms, echo time = 2.53 ms, inversion time = 900 ms, field of view = 192 mm and slice thickness = 1 mm. The voxel size was 1 mm × 1 mm × 1 mm.

### Data processing

For image processing, we used SPM12 software (Statistical Parametric Mapping; Wellcome Trust Centre for Neuroimaging, London, UK; http://www.fil.ion.ucl.ac.uk/spm) running on MATLAB R2010a (MathWorks, Natick, MA, USA). Images of each patient were spatially normalised to the Montreal Neurological Institute space. T1-weighted images were segmented into grey matter, white matter, cerebrospinal fluid (CSF), bone, fat and air. Grey matter images were modulated and smoothed with a Gaussian kernel of 8 mm.

### Voxel-based morphometry

Atrophy of grey matter was analysed using voxel-based morphometry (VBM) in SPM12 in an exploratory whole-brain analysis. First, two-sample one-tailed *t* tests between the HC group and the DLB group and between the HC group and the AD group were performed in order to confirm that brain atrophy in the DLB group and the AD group was congruent with previous reports in the literature. Second, we performed an analysis in the DLB group to search for neural correlates of ToM impairment. For this purpose, we performed a one-sample, one-tailed *t* test to investigate the correlation between each ToM score and grey matter volume, and a two-sample, one-tailed *t* test to compare patients with DLB who were impaired with those who were non-impaired for each ToM test. Age and global grey matter volume were considered as nuisance covariates for all of the analyses. Statistical maps were thresholded at *P* < 0.001, uncorrected, and with a minimum cluster size of 25 voxels. Statistical maps were analysed with xjView (http://www.alivelearn.net/xjview8/), which enabled us to identify the brain regions that were associated with the detected clusters.

### Statistical analysis

IBM SPSS version 21.0 software (IBM, Armonk, NY, USA) was used for further statistical evaluation as required. Where appropriate, differences in demographic and clinical data were assessed using parametric (analysis of variance, *t* tests) and non-parametric tests (Kruskal-Wallis test) with post hoc analysis. For categorical measures, χ^2^ tests were applied. Correlations between clinical data, neuropsychological tests and ToM tests were assessed using Spearman’s test. For each test statistic, *P* < 0.05 was regarded as significant.

## Results

### Population characteristics

Ninety-six participants were recruited to perform ToM tasks. Twenty-eight patients were excluded for the following reasons: too severe stage of the disease (*n* = 9), presence of another neurological or psychiatric disease (*n* = 10), possible DLB or AD but without criteria for probable DLB or AD (*n* = 5), association of DLB and AD (*n* = 1), subjective memory complaint without objective impairments on neurological tests (*n* = 2) and insufficient knowledge of French (*n* = 1). Four healthy subjects were excluded because of psychiatric disorder (*n* = 1), incomplete file (*n* = 1), presence of hallucinations (*n* = 1) and presence of parkinsonism (*n* = 1). Ultimately, 64 participants were included in the study: 33 patients in the DLB group, 15 patients in the AD group and 16 elderly healthy subjects in the HC group.

There were no significant differences between the DLB, AD and HC groups for age, sex, handedness or level of education (all *P* > 0.05). The three groups’ demographic and clinical characteristics are presented in Table 1. There were no significant differences between the DLB and AD groups for disease duration, mean MMSE score or mean instrumental activities of daily living (IADL) score. Eighty-two percent of patients with DLB and 80 % of patients with AD had a lumbar puncture. AD biomarkers were always negative in the patients with DLB and always positive in the patients with AD. Fourteen patients with DLB underwent DatScan SPECT, and dopaminergic denervation was found in 64 % of cases.

**Table 1:** Clinical and demographic features of patients with dementia with Lewy bodies, patients with Alzheimer’s disease and healthy elderly control subjects

	HC (*n* = 16)	DLB (*n* = 33)	AD (*n* = 15)	Test statistic, *P* value	Post hoc analysis
Sex, M/F	7/9	16/17	8/7	χ^2^ = 0.097, *P* = 0.952	
Age, yr^a^	68.3 (10.5)	68 (8.4)	70.9 (11.1)	F = 0.491, *P* = 0.614	
Handedness, R/L	15/1	30/3	14/1	χ^2^ = 0.156, *P* = 0.925	
Education, yr^a^	11.9 (3.2)	12.4 (3.2)	13.5 (3.6)	F = 0.882, *P* = 0.419	
Disease duration, yr^a^		4.6 (4.2)	3.6 (1.8)	F = 0.715, *P* = 0.402	
MMSE^a^	29.3 (0.9)	27.2 (1.8)	27 (2.6)	H = 17.833, *P* < 0.000	HC > DLB and AD
IADL on scale of 8^a^	7.8 (0.4)	7.1 (1.3)	7.6 (0.8)	H = 3.671, *P* = 0.160	
Hallucinations	0 %	66.6 %	0 %	χ^2^ = 31.492, *P* < 0.000	
Fluctuations					
Mayo Clinic Fluctuation Scale	37.5 %	93.9 %	26.7 %	χ^2^ = 26.810, *P* < 0.000	
Clinician Assessment of Fluctuation	0.56 (2.25)	3.78 (3.1)	0.38 (1)	H = 22.050, *P* < 0.000	DLB > HC and AD
Parkinsonism					
Akinesia (0/1/2/3/4)	16/0/0/0/0	11/18/4/0/0	15/1/0/0/0	H = 27.189, *P* < 0.000	DLB > HC and AD
Rigidity (0/1/2/3/4)	16/0/0/0/0	4/25/3/1/0	10/5/0/0/0	H = 35.112, *P* < 0.000	DLB > HC and AD
Tremor at rest (0/1/2/3/4)	16/0/0/0/0	20/12/0/0/0	13/2/0/0/0	H = 9.031, *P* = 0.011	DLB > HC
Treatments					
Cholinesterase inhibitor	0 %	28.1 %	41.7 %	χ^2^ = 7.535 *P* = 0.023	
Neuroleptics	0 %	6.3 %	0 %	χ^2^ = 1.810, *P* = 0.404	
l-dopa or dopaminergic agonists	6.3 %^b^	43.8 %	0 %	χ^2^ = 11.611, *P* = 0.003	
Cerebrospinal fluid					
Normal/abnormal		27/0	0/12	χ^2^ = 40, *P* < 0.000	
Aβ_42_^a^		943.1 (334.5)	533.3 (306.9)	F = 12.369, *P* = 0.001	DLB > AD
Tau^a^		303.1 (110.1)	643.1 (305.7)	F = 27.135, *P* < 0.000	AD > DLB
p-Tau^a^		46.3 (14.3)	86.7 (26.6)	F = 38.712, *P* < 0.000	AD > DLB
IATI^a^		1.7 (0.5)	0.6 (0.3)	F = 35.606, *P* < 0.000	DLB > AD
Hippocampi atrophy					
Right (0/1/2/3/4)	4/9/2/0/0	11/10/5/1/0	0/3/6/0/0	H = 8.030, *P* = 0.018	AD > HC and DLB
Left (0/1/2/3/4)	6/7/2/0/0	13/8/5/1/0	0/5/3/1/0	H = 6.590, *P* = 0.037	AD > DLB

*Aβ*_*42*_ amyloid-β_42_ *AD* Alzheimer’s disease, *HC* healthy control subjects, *DLB* dementia with Lewy bodies, *MMSE* Mini Mental State Examination, *IADL* Instrumental Activities of Daily Living, *IATI* INNOTEST Amyloid Tau Index (Fujirebio, Ghent, Belgium), *F* analysis of variance, *H* Kruskal-Wallis test, *p-tau* phosphorylated tau

^a^Mean (standard deviation)

^b^One healthy control subject had dopaminergic agonist treatment for restless legs syndrome

The results of the general neuropsychological tests are presented in Table 2. Patients with DLB were in a stage of mild cognitive impairment, but they performed poorly compared with the HC group in the majority of tasks. The mean MMSE scores were 27.2 for patients with DLB, 27 for patients with AD and 29.3 for HC. Patients with DLB had significantly more memory impairments than HC (FCSRT and DMS48) and more difficulty with executive, speed processing, praxis and attentional tasks (TMT A and B, DSST, digit span and lexical evocation). The DLB and AD groups performed similarly on all these tests except the FCSRT, on which patients with AD performed worse than patients with DLB, and also abstract praxis, on which the DLB group performed worse than the AD group.

**Table 2:** Neuropsychological features of patients with dementia with Lewy bodies, patients with Alzheimer’s disease and healthy elderly control subjects

	HC (*n* = 16)	DLB (*n* = 33)	AD (*n* = 15)	Test statistic, *P* value	Post hoc analysis
FAB on scale of 18^a^	17.3 (1.2)	15.3 (2.2)	15 (3.2)	H = 13.343, *P* = 0.001	HC > DLB and AD
FCSRT					
IR on scale of 16^a^	15.8 (0.4)	14.8 (1.4)	13.5 (2)	H = 18.429, *P* < 0.000	HC > DLB and AD, DLB > AD
FR on scale of 48^a^	31.3 (3.9)	22.6 (7.9)	9.4 (7)	H = 36.616, *P* < 0.000	HC > DLB and AD, DLB > AD
TR on scale of 48^a^	47.3 (0.9)	43 (6.8)	25.5 (12.3)	H = 32.488, *P* < 0.000	HC > DLB and AD, DLB > AD
CFR on scale of 16^a^	12.6 (1.7)	8.5 (3.8)	3.3 (4)	H = 26.461, *P* < 0.000	HC > DLB and AD, DLB > AD
CTR on scale of 16^a^	15.9 (0.3)	14.8 (2)	8.8 (4.9)	H = 26.548, *P* < 0.000	HC and DLB > AD
DMS48					
Set 1,^a^ %	99 (2)	93 (6)	88 (11)	H = 13.752, *P* = 0.001	HC > DLB and AD
Set 2,^a^ %	99 (1)	93 (7)	83 (10)	H = 19.771, *P* < 0.000	HC > DLB and AD
Rey-Osterrieth complex figure test^a^	34.5 (1.6)	29.6 (7.9)	31.7 (4.2)	H = 6.088, *P* = 0.048	HC > DLB
VOSP					
Number localisation^a^	10.1 (2.7)	8.2 (2.2)	8.3 (2.3)	H = 5.526, *P* = 0.063	
Cube analysis^a^	10 (2.1)	9 (1.5)	7.9 (3.4)	H = 2.677, *P* = 0.262	
TMT A^a^	42.7 (13.9)	64.3 (27.7)	53.7 (15.3)	H = 9.09, *P* = 0.011	DLB > HC
TMT B^a^	97.9 (34.1)	164.2 (89.8)	149.5 (73.5)	H = 8.994, *P* = 0.011	DLB > HC
DSST on scale of 19^a^	11.4 (2.3)	7.7 (2.3)	10.3 (3)	H = 17.972, *P* < 0.000	HC > DLB
Digit span					
Direct^a^	8.8 (2.2)	6.8 (1.9)	7.7 (2.6)	H = 4.499, *P* = 0.105	
Indirect^a^	5.8 (1.8)	4.4 (1.5)	4.5 (1.4)	H = 7.781, *P* = 0.02	HC > DLB
Lexical evocation					
Formal^a^	24.6 (8.6)	16 (7.7)	22.7 (7.1)	H = 11.814, *P* = 0.003	HC > DLB
Semantic^a^	37 (7.9)	23.7 (7.5)	21.6 (9.5)	H = 23.494, *P* < 0.000	HC > DLB and AD
DO80^a^	79.6 (0.6)	77.1 (3.5)	71.2 (20.3)	H = 8.655, *P* = 0.013	HC > DLB and AD
Praxis					
Symbolic^a^	4.9 (0.5)	4.7 (0.5)	5.4 (1.6)	H = 4.104, *P* = 0.128	
Action^a^	9.9 (0.3)	9 (1.3)	9.4 (0.8)	H = 6.761, *P* = 0.034	HC > DLB
Abstract^a^	7.9 (0.5)	6.6 (1.7)	7.9 (0.3)	H = 14.780, *P* = 0.001	HC and AD > DLB

*AD* Alzheimer’s disease, *CFR* cued free recall, *CTR* cued total recall, *DMS* Delayed Matching to Sample, *DLB* dementia with Lewy bodies, *DO* Dénomination Orale (an oral naming test), *DSST* Digit Symbol Substitution Test, *FAB* Frontal Assessment Battery, *FCSRT* Free and Cued Selective Reminding Test, *FR* free recall, *HC* healthy control subjects, *IR* immediate recall, *TMT* Trail Making Test, *TR* total recall, *VOSP* Visual Object and Space Perception, *H* Kruskal-Wallis test

^a^Mean (standard deviation)

### ToM tasks

Table 3 displays data for ToM tasks.

**Table 3:** Theory of mind tests of patients with dementia with Lewy bodies, patients with Alzheimer’s disease and healthy elderly control subjects

	HC (*n* = 16)	DLB (*n* = 33)	AD (*n* = 15)	Test statistic, *P* value	Post hoc analysis
Ekman’s Facial Emotion Recognition test					
Impaired subjects	12.5 %	28.6 %	23.1 %	χ^2^ = 0.830, *P* = 0.660	
Total score on scale of 35	28.6 (3.2)	27.1 (3.3)	26.6 (2.8)	H = 2.39, *P* = 0.303	
Happiness on scale of 5	5 (0)	4.8 (0.4)	4.8 (0.4)	H = 3.244, *P* = 0.197	
Fear on scale of 5	2.1 (1.3)	2.1 (1.3)	1.9 (1.6)	H = 0.295, *P* = 0.863	
Disgust on scale of 5	4.3 (0.8)	4 (1)	4.3 (1.3)	H = 3.337, *P* = 0.189	
Anger on scale of 5)	3.6 (1.3)	3.9 (1.1)	3.6 (1.3)	H = 0.344, *P* = 0.842	
Surprise on scale of 5	4.9 (0.3)	4.1 (0.7)	4.1 (0.6)	H = 15.573, *P* = 0.001	HC > DLB and AD
Sadness on scale of 5	3.7 (1.3)	3.8 (1.1)	3.5 (1.4)	H = 0.174, *P* = 0.917	
Neutral on scale of 5	4.9 (0.3)	4.5 (1)	4.5 (0.7)	H = 3.774, *P* = 0.151	
Reading the Mind in the Eyes test					
Impaired subjects	14.3 %	48.4 %	33.3 %	χ^2^ = 4.066, *P* = 0.131	
Total score on scale of 36	23.9 (2.8)	20.6 (4.2)	21.6 (3.4)	H = 8.425, *P* = 0.015	HC > DLB
Faux Pas Recognition test					
Impaired subjects	12.5 %	42.3 %	43.9 %	χ^2^ = 5.301 *P* = 0.071	
Faux pas stories on scale of 30	23.8 (4.2)	19.3 (5.3)	20.6 (6)	H = 6.817, *P* = 0.033	HC > DLB
Corrects hits on scale of 5	4.8 (0.4)	4.3 (1)	4.4 (0.9)	H = 2.61, *P* = 0.271	
Question 3, %	84.1 (18.4)	76.4 (25.3)	75.5 (16.3)	H = 1.761, *P* = 0.415	
Question 4, %	48.1 (24.3)	35.5 (26.5)	33.6 (26.4)	H = 3.091, *P* = 0.213	
Intentionality, %	80.9 (22.2)	82.5 (20.1)	69.2 (28.3)	H = 2.611, *P* = 0.271	
Emotional attribution, %	89.1 (15.4)	71.4 (26.4)	83 (17.5)	H = 5.493, *P* = 0.064	
Correct rejection on scale of 10	9.6 (1.1)	8.9 (2)	8.7 (2.2)	H = 2.04, *P* = 0.361	
CQ on scale of 20	19.9 (0.3)	19.2 (1.7)	19.1 (1)	H = 5.868, *P* = 0.053	
Mini-SEA on scale of 30	24.7 (2.4)	21.9 (2.9)	22.7 (2.2)	H = 9.682, *P* = 0.008	HC > DLB

*AD* Alzheimer’s disease, *DLB* dementia with Lewy bodies, *HC* healthy control subjects, *CQ* control questions, *Mini-SEA* Social cognition and Emotional Assessment, *H* Kruskal-Wallis test

Data are expressed as percentage or mean (standard deviation)

#### Ekman’s Facial Emotion Recognition test

We found no statistical difference between the three groups for Ekman’s Facial Emotion Recognition test. A similar percentage of subjects failed this test in the two groups of patients (28.6 % in the DLB group versus 23.1 % in the AD group and 12.5 % in HC subjects; *P* = 0.66). Mean scores were similar between the three groups (27.1 in the DLB group versus 26.6 in the AD group and 28.6 in the HC group; *P* = 0.303). All the subjects had difficulty in identifying fear, anger and sadness, in the same proportions (*P* > 0.05). The only difference between the three groups concerned the recognition of surprise (4.1 in the DLB group and the AD group versus 4.9 in the HC group, *P* = 0.001).

#### Reading the Mind in the Eyes test

We found a significant difference between patients with DLB and the HC group for the mean score on this test (20.6 in the DLB group versus 23.9 in the HC group; *P* = 0.015). Forty-eight percent of patients with DLB failed this test versus 14 % of the HC group. Thirty-three percent of patients with AD failed this test, but there were no significant differences between the AD group and the HC group or between the AD group and the DLB group.

#### Faux Pas Recognition test

According to the mini-SEA standardized score for the FPR test, 42 % of patients with DLB and 43 % of patients with AD failed this test versus 12 % of HC (*P* = 0.071). Patients with DLB performed significantly worse than HC for faux pas stories (*P* = 0.033). There were no significant differences between the AD group and the HC group or between the AD group and the DLB group. No significant difference was observed between the three groups for the number of correct hits (*P* = 0.271), question 3 (*P* = 0.415) and question 4 (*P* = 0.213), intentionality (*P* = 0.271), or emotional attribution (*P* = 0.064). Patients with DLB performed worse than the other groups for this last question, but the difference was not significant. Patients with DLB and patients with AD correctly rejected the control stories. The comprehension of stories was preserved, with high scores for control questions in the three groups (*P* = 0.053).

#### Mini-Social cognition and Emotional Assessment score

A significant difference was found between the DLB group and the HC group for the global mini-SEA score (*P* = 0.008) (Table 3). This score is based on Ekman’s Facial Emotion Recognition test score and the composite score of the FPR test. Patients with AD had a lower score than HC, but the difference was not significant. There was no significant difference between the DLB group and the AD group.

We found a significant correlation between education level and scores on Ekman’s Facial Emotion Recognition test, the RME test and the FPR test (all *P* < 0.05). The score on the RME test was correlated with executive function (FAB, TMT B and semantic lexical evocation), the Rey-Osterrieth complex figure test score and the IADL score. We did not find any correlations between the RME test, the FPR test, the mini-SEA score and either age or disease duration. Ekman’s Facial Emotion Recognition test and FPR test scores were not correlated with any of the neuropsychological tests (Table 4).

**Table 4:** Spearman’s correlations between clinical and neuropsychological data and performances on the theory of mind tasks

	Ekman’s Facial Emotion Recognition test	RME test	FPR test	Mini-SEA
Age	−0.408	−0.351	0.072	−0.026
Education level	0.527^a^	0.265	0.410^a^	0.527^a^
Disease duration	−0.125	0.007	0.198	−0.017
IADL	0.433	0.423^a^	0.198	0.210
MMSE	0.216	0.243	0.226	0.262
FAB	0.365	0.480^b^	0.193	0.331
Rey-Osterrieth complex figure test	0.312	0.410^a^	0.770	0.226
Number localisation	0.358	0.241	0.177	0.324
Cube analysis	0.189	0.213	0.023	0.337
TMT A	0.102	−0.231	−0.220	−0.175
TMT B	−0.230	−0.549^b^	−0.024	−0.160
Indirect digit span	0.088	0.140	−0.270	−0.138
Phonemic lexical evocation	0.276	0.287	0.269	0.248
Semantic lexical evocation	0.295	0.403^a^	0.254	0.198

*IADL* Instrumental Activities of Daily Living test, *FAB* Frontal Assessment Battery, *FPR* Faux Pas Recognition, *MMSE* Mini Mental State Examination, *RME* Reading the Mind in the Eyes, *Mini-SEA* mini-Social cognition and Emotional Assessment, *TMT* Trail Making Test

^a^ *P* < 0.05

^b^ *P* < 0.005

### VBM analysis

#### Comparison of atrophy between patients with DLB, patients with AD and HC group

**Fig. 1:**
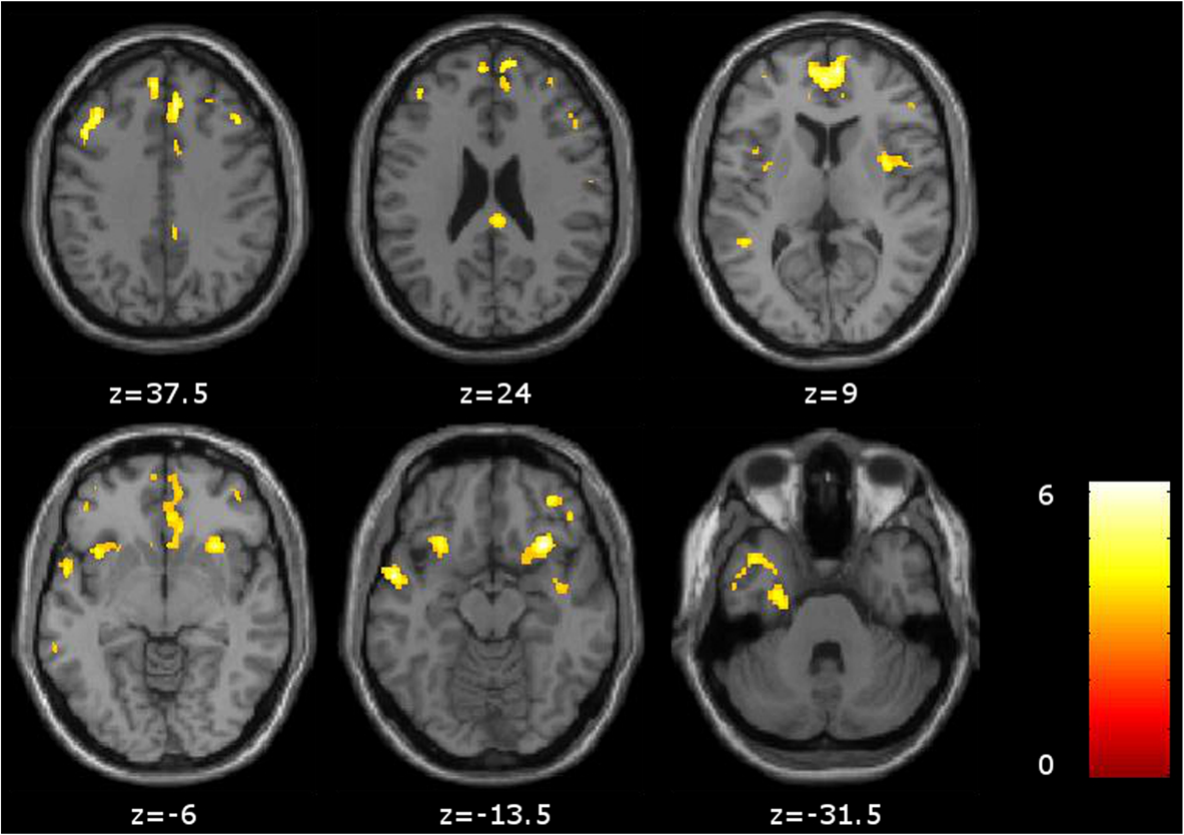
Voxel-based morphometric analysis of global atrophy in the dementia with Lewy bodies group compared with the healthy control group. Comparison between the dementia with Lewy bodies group and the healthy control group shows relative grey matter atrophy in frontal, temporal and insular regions (*P* < 0.001, uncorrected, minimum cluster size of 25 voxels)

VBM analysis revealed some regions of atrophy in the DLB group, compared with HC, in the frontal lobe (medial, middle and inferior frontal gyri; anterior cingulate cortex; and orbital gyrus), the temporal lobe (bilateral superior and left inferior temporal gyri) and the bilateral insula (*P* < 0.001, uncorrected, minimum cluster size of 25 voxels) (Fig. 1 and Additional files 1 and 2).

**Fig. 2:**
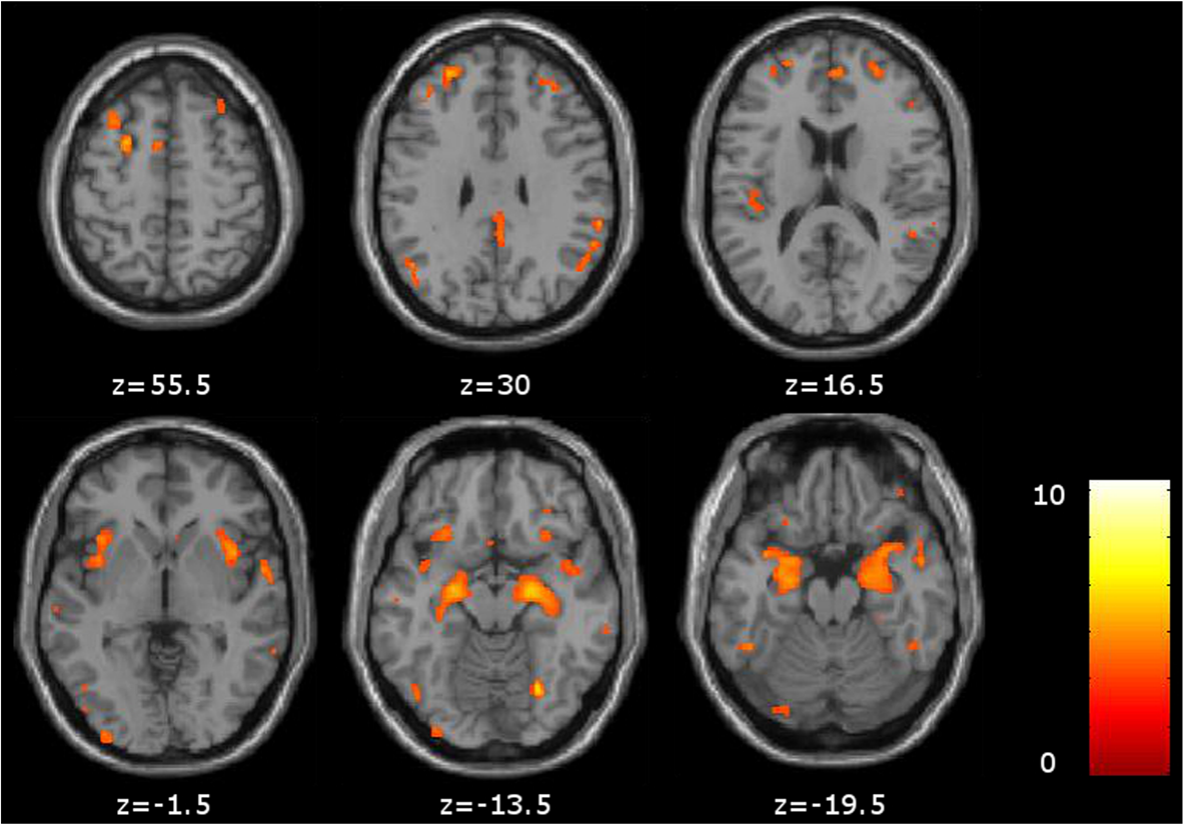
Voxel-based morphometric analysis of global atrophy in the Alzheimer’s disease group compared with the healthy control group. Comparison between the Alzheimer’s disease group and the healthy control group shows relative grey matter atrophy in medial temporal lobes and frontal regions (*P* < 0.001, uncorrected, minimum cluster size of 25 voxels)

Patients with AD were particularly atrophied in the bilateral medial temporal lobe (fusiform and parahippocampal gyri, hippocampus) compared with HC (*P* < 0.05, family-wise error–corrected, minimum cluster size of 25 voxels). They also presented more atrophy than HC in the frontal lobe (superior, middle and medial frontal gyri and cingulate gyrus), the right superior temporal gyrus and the right insula (*P* < 0.001, uncorrected, minimum cluster size of 25 voxels) (Fig. 2 and Additional files 1 and 2).

Compared with the DLB group, the AD group was more atrophied in the left middle frontal gyrus, the bilateral parietal lobe (superior and inferior parietal gyrus, precuneus), the right cuneus and the left lingual gyrus, and especially in the bilateral medial temporal lobe (fusiform gyrus, amygdala, hippocampus and parahippocampal gyrus). Compared with the AD group, the DLB group was more atrophied in the left cingulate gyrus and the right middle frontal gyrus (*P* < 0.001, uncorrected, minimum cluster size of 25 voxels) (Additional files 1 and 2).

#### Correlation analysis for Ekman’s Facial Emotion Recognition test

**Fig. 3:**
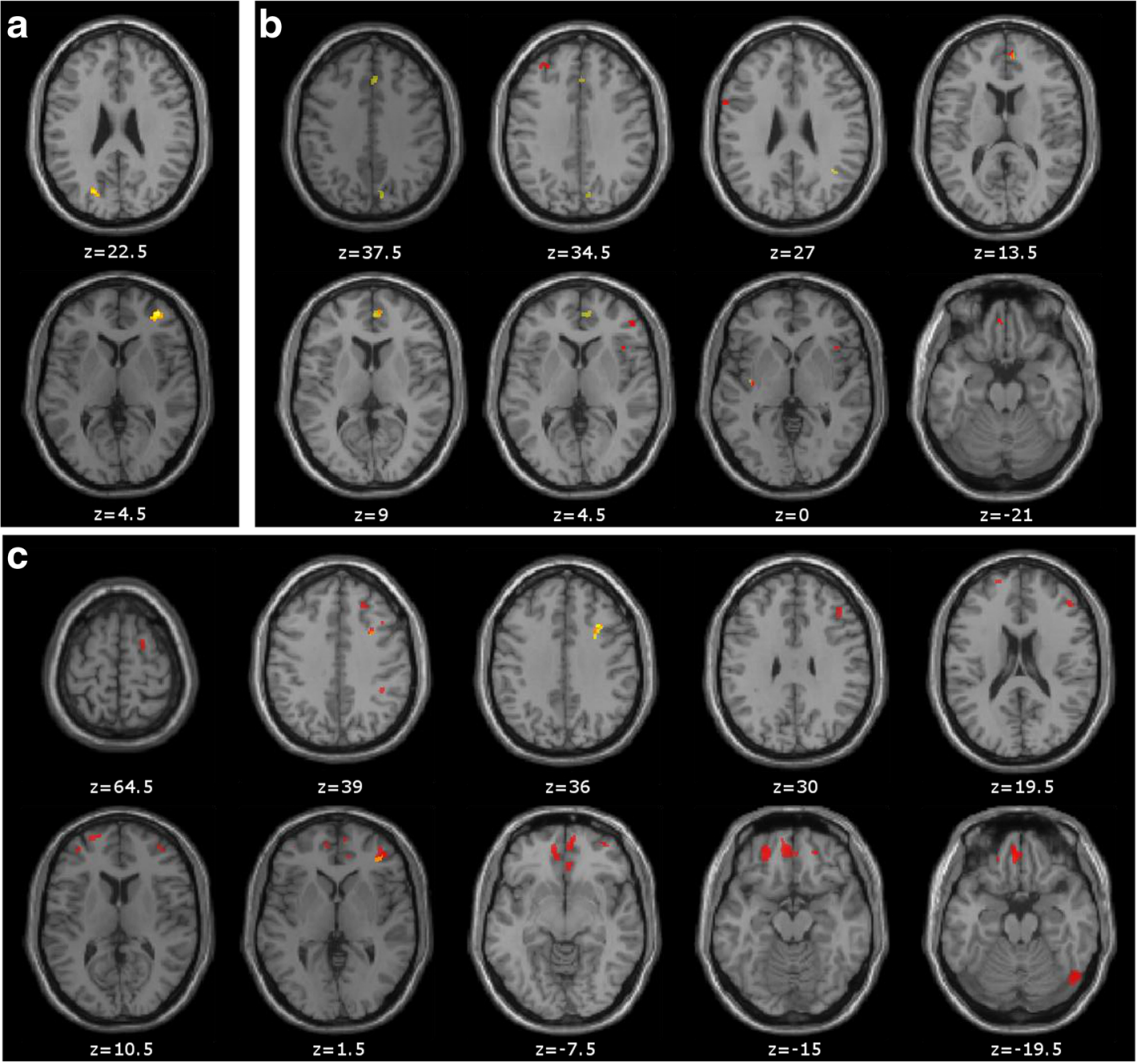
Voxel-based morphometric analysis for correlations between grey matter volume and theory of mind tasks in the dementia with Lewy bodies (DLB) group, and group analysis between impaired and non-impaired patients with DLB. **a** Ekman’s Facial Emotion Recognition test: correlations between scores and grey matter atrophy in the DLB group. **b** Faux Pas Recognition test: correlations between scores and grey matter atrophy in the DLB group (*yellow*) and relative atrophy in impaired patients with DLB compared with non-impaired patients (*red*). **c** Reading the Mind in the Eyes test: correlations between scores and grey matter atrophy in the DLB group (*yellow*) and relative atrophy in impaired patients with DLB compared with non-impaired patients (*red*). *P* < 0.001, uncorrected, minimum cluster size of 25 voxels

In the DLB group, impairment on Ekman’s Facial Emotion Recognition test was correlated with atrophy in the right middle frontal gyrus (Brodmann’s area [BA] 10) and the left precuneus (BA 31) (*P* < 0.001, uncorrected, minimum cluster size of 25 voxels) (Table 5 and Fig. 3).

**Table 5:** Voxel-based morphometric analysis for correlations between grey matter volume and theory of mind tasks in the dementia with Lewy bodies group, and group analysis between impaired and non-impaired patients with dementia with Lewy bodies

Anatomical regions	BA	R/L	MNI coordinates			T value	Cluster size
			*x* axis	*y* axis	*z* axis		
Ekman’s Facial Emotion Recognition test							
Correlations							
Middle frontal gyrus	10	R	36	51	7	8.27	130
Precuneus	31	L	−21	−73	25	6.29	37
RME test							
Correlations							
Middle frontal gyrus	9	R	31	18	36	6.16	99
Inferior frontal gyrus	10	R	39	39	−0	4.41	28
Group analysis							
Superior frontal gyrus	9	R	26	35	39	5.30	36
	10	L	−16	62	15	5.60	177
	11	R	22	47	−15	4.33	27
Middle frontal gyrus	6	R	20	5	67	5.18	117
	10	R	40	50	6	7.10	283
	11	L	−24	50	−14	6.11	168
	11	R	38	54	−8	5.20	32
	46	R	42	29	27	4.73	184
	46	L	−34	51	7	4.56	47
Orbital gyrus	11	L	−3	45	−20	6.46	1396
Inferior parietal lobule	40	R	39	−42	40	4.07	33
Fusiform gyrus	19	R	52	−66	−23	6.84	322
FPR test							
Correlations							
Medial frontal gyrus	10	R	8	51	10	9.42	129
Cingulate gyrus	32	R	3	29	36	5.33	51
Supramarginal gyrus	40	R	37	−52	25	5.55	28
Precuneus	7	R	9	−76	36	5.16	47
Insula	13	L	−38	−7	−8	4.67	42
Group analysis							
Superior frontal gyrus	9	L	−27	42	33	6	42
Medial frontal gyrus	10	R	8	51	10	4.41	50
Inferior frontal gyrus	46	R	49	42	6	5.64	25
	9	L	−60	9	28	5.15	42
Orbital gyrus	11	L	−3	45	−21	5.88	27
Insula	13	L	−38	−6	−9	5.94	41
	13	R	40	20	3	5.70	29

*BA* Brodmann’s area, *FPR* Faux Pas Recognition, *R/L* right/left, *MNI* Montreal Neurological Institute, *RME* Reading the Mind in the Eyes

*P* < 0.001, uncorrected, minimum cluster size of 25 voxels

T value: test statistic for t-tests

Owing to the limited number of MRI images for impaired patients with DLB for this test (*n* = 4), no significant results were found in the group analysis comparing impaired and non-impaired patients in the DLB group.

#### Correlation analysis and group analysis for the RME test

Impairment on the RME test in the DLB group was correlated with diminished volume of the right middle (BA 9) and inferior frontal (BA 10) gyri (*P* < 0.001, uncorrected, minimum cluster size of 25 voxels) (Table 5 and Fig. 3).

Group analysis comparing impaired and non-impaired patients with DLB revealed atrophy in impaired patients in the bilateral superior and middle frontal gyri (BA 6, 9, 10, 11 and 46), the left orbital gyrus (BA 11), the right inferior parietal lobule (BA 40) and the right fusiform gyrus (BA 19) (*P* < 0.001, uncorrected, minimum cluster size of 25 voxels) (Table 5 and Fig. 3).

#### Correlation analysis and group analysis for the FPR test

Impairment on the FPR test in the DLB group was correlated with atrophy in the right medial frontal gyrus (BA 10), the right cingulate gyrus (BA 32), the right supramarginal gyrus (BA 40), the right precuneus (BA 7) and the left insula (BA 13) (*P* < 0.001, uncorrected, minimum cluster size of 25 voxels) (Table 5 and Fig. 3). Group analysis comparing impaired and non-impaired patients with DLB showed diminished volume in impaired patients in frontal regions (superior, medial and inferior frontal gyri), the left orbital gyrus and the left insula (*P* < 0.001, uncorrected, minimum cluster size of 25 voxels) (Table 5 and Fig. 3).

## Discussion

### ToM impairments in dementia with Lewy bodies

This study is the first to assess ToM performance in patients with DLB. The results confirm the hypothesis of ToM impairments in patients with DLB, with deficits on the RME test and on the FPR test. The patients with DLB in our study were mildly impaired, some with normal activity of daily living, with a mean MMSE score of 27 and relatively preserved performance on other neuropsychological tasks. Thus, ToM seems to be impaired at the early stage of the DLB.

Patients with DLB had preservation of low-demand affective ToM tasks such as Ekman’s Facial Emotion Recognition test but were impaired for high-demand affective ToM tasks such as the RME test. In Ekman’s Facial Emotion Recognition test, patients and HC were similarly impaired in recognizing negative emotions such as fear, anger, disgust and sadness. In their meta-analysis, Ruffman et al. [47] showed that older healthy adults performed worse than young adults in identifying these negative emotions. Their finding may explain why all three groups in our study were impaired for the recognition of negative emotions. On the RME test, nearly 50 % of our patients with DLB failed versus only 15 % of HC; therefore, the deficit was not due to ageing, and DLB does indeed seem to cause impairments of high-demand affective ToM. The difference in score between the AD group and the HC group was not significant, but we were not able to show a significant difference between AD and patients with DLB.

Patients with DLB were also impaired for cognitive ToM tasks such as the FPR test. Forty-two percent of the patients with DLB failed on this test. They performed worse than the HC group on faux pas stories, which reflect the detection and explanation of faux pas. The score of the AD group was better than that of the DLB group but lower than that of the HC group, though neither of these differences reached significance. The most marked deficiency in the DLB group, compared with the HC and AD groups, was on the question about emotional attribution, which assesses the affective component of ToM. Impairments on this test were not caused by an inability to understand the stories as all the subjects performed well on the control questions.

Thus, both affective and cognitive components were impaired in DLB, patients having difficulty in interpreting the intentions of others, recognising complex feelings and imagining another’s feelings in a story. The distinction between these two components of ToM is interesting because different pathways would be implicated for each of them [5, 6, 48, 49]. In their review, Poletti et al. [4] proposed that each neurodegenerative disease would lead to a specific pattern of ToM deficits depending on its neuropathological course and these different patterns could help physicians to diagnose and distinguish between these diseases.

The majority of studies that assessed ToM in AD found impairments on high-cognitive-demanding ToM tasks whereas low-cognitive-demanding ToM tasks were preserved [9, 11, 50–54]. As we observed in our study, Gregory et al. [9] and Narme et al. [55] found lower scores in patients with AD than in healthy control subjects for the FPR test, but without significant difference. For the RME test, the data are more heterogeneous. Gregory et al. [9] found that the performance of patients with AD was preserved for this test, but Castelli et al. [53] and Laisney et al. [54] found the opposite. In these latter two studies, patients with AD were older and had lower mean scores on the MMSE than those in the study by Gregory et al. [9], which might explain the different performance on the RME test. In our study, patients with AD were more similar to the patients with AD in Gregory et al.’s study. Overall, the ToM capacities of patients with AD in the early stage of the disease seem to be preserved, because they seem to experience difficulties only with high-demand ToM tasks. In DLB, ToM deficits seem to be more global and occur earlier than those reported in AD, but we did not find any significant differences between these two groups.

The relationship between ToM and executive function is a matter of debate. In their review of the literature, Aboulafia-Brakha et al. [56] showed a congruent association between ToM and executive function in 64 % of studies and incongruent associations in 29 % of studies. In this review, both FPR and RME tests were strongly correlated with executive function. In our study, we found some correlations between executive function tests and the RME test but not with Ekman’s Facial Emotion Recognition test or the FPR test. In the DLB group, ToM and other cognitive abilities (executive and visuoperceptive functions) seemed to be relatively independent. Even if ToM can be linked with executive function in some circumstances, it seems to be a distinct cognitive function and its exploration would be useful in clinical practice. However, education level plays a role in success on ToM tasks.

The social impact of ToM impairments needs to be investigated further. Few studies have been done on the clinical implications of ToM. However, Gregory et al. [9] found a correlation between ToM impairments and the Neuropsychiatric Inventory score, which reflects behavioural disorders. Deficits of ToM in DLB could explain social interaction disorders such as loss of empathy, egocentricity or aggressiveness.

### Neural correlates of ToM impairments

Compared with HC, patients with DLB were atrophied in the frontal and lateral temporal lobes and in the bilateral insula. These results are consistent with previous data [57–59], particularly for the insula. Indeed, in their meta-analysis, Zhong et al. [59] showed that the insula is a core region for atrophy in DLB and could have a role in autonomic dysfunction. As expected, the patients with AD in our study were more atrophied in the medial temporal lobes than were the HC and patients with DLB. These patterns of atrophy demonstrate the homogeneity of each group.

VBM analysis showed correlations between ToM tasks and cerebral atrophy in different cerebral regions. Prefrontal cortex atrophy was correlated with impairments on the three ToM tests; specific cerebral regions were then found for each test. The prefrontal cortex is known for its role in executive function, such as attention, planning, decision-making and working memory [60]. This region is classically associated with ToM [7, 46, 61–63]. In their review, Carrington and Bailey [3] reported an involvement of the medial prefrontal cortex and orbitofrontal cortex in 93 % of studies and an involvement of the lateral prefrontal cortex in 35 % of studies. According to lesion and neuroimaging studies, the medial prefrontal cortex is implicated in affective ToM [5] while the lateral prefrontal cortex is implicated in cognitive ToM [6]. In our study, RME impairments were particularly correlated with atrophy in the orbitofrontal cortex. This region has often been reported to be involved in affective ToM [64–66]. Its role in regulation of emotions is known, and patients with lesions in the orbitofrontal cortex have social behaviour impairments. Hynes et al. [64] stated that the orbitofrontal cortex has a role in the automatic response to a social stimulus, without explicit judgement about this stimulus, and allows us to adapt our behaviour. In our study, the FPR test was also correlated with the medial prefrontal cortex and orbitofrontal cortex. Even if the FPR test assesses cognitive ToM, some studies found a link between the FPR test and the medial prefrontal cortex [48, 67, 68]. Moreover, patients with DLB failed especially on the last question of the FPR test, concerning emotional attribution. The atrophy in the medial prefrontal cortex may explain the difficulty in this empathic question, and consequently the FPR test impairments.

The inferior frontal cortex is part of the mirror neuron system and is engaged in both the execution of actions and the observation of these actions performed by someone else. According to the theory of simulation, other people’s mental states are represented by simulating their states with resonant states of one’s own [69]. This process implies mirror neurons and explains the role of the inferior frontal cortex in the RME and FPR tests.

The RME and FPR tests were also correlated with regions of the right TPJ (inferior parietal lobule). This region is strongly associated with ToM for both the affective and the cognitive components [3, 70]. Uddin et al. [71] demonstrated the role of the right TPJ in self–other distinction. In their study, subjects failed to discriminate self-faces and other familiar faces when repetitive transcranial magnetic stimulation was applied over this region. Decety and Lamm [72] performed a meta-analysis to attempt to understand the role of the TPJ in ToM. In their view, the activation in the TPJ during ToM tests relies on a ‘lower-level computational mechanism involved in generating, testing, and correcting internal predictions about external sensory events’ and is not due to a specific role in ToM.

The precuneus, which correlated with FPR and Ekman’s Facial Emotion Recognition tests in our study, is a core region of ToM [3, 70]. It is known for its role in visuospatial perception and episodic memory. According to Cavanna and Trimble [73], the precuneus plays a role in ToM by using mental imagery to represent the perspective of another person.

Patients with RME test impairments had relatively more atrophy in the fusiform gyrus. This region was reported to be related to face and object perception [74]. Furthermore, Gallagher et al. [75] found an association between the fusiform gyrus and understanding the meaning of visual jokes. In a similar manner, Baglio et al. [63] studied performances on the RME test in patients with amnestic mild cognitive impairment and found activation of the bilateral fusiform gyrus during the test. ToM capacities required facial examination mediated by the fusiform gyrus.

Patients with FPR test impairments had relatively more atrophy in the bilateral insula. The role of the insula in emotional response is well known. Bertoux et al. [46] also found a correlation between the insula and the FPR test, particularly with the explanation of faux pas. They supposed that this region was involved in impairment of the last question, about emotional attribution. This hypothesis is congruent with our findings because the results for this question were the most impaired in the DLB group compared with HC.

To summarise, ToM capacities are based not on a single cerebral region but on a neuronal network, including prefrontal regions and the TPJ, precuneus and insula. Our findings in patients with DLB are consistent with those reported in healthy subjects, psychiatric patients and patients with other neurodegenerative cognitive diseases, and they support the existence of such a network. Further studies, including functional connectivity (functional and arterial spin labelling MRI) and anatomical connectivity (diffusion tensor imaging), are now needed to better characterize this network.

Our study has some limitations. The main limitation is that patients with DLB were included on the basis of clinical criteria. Today, the gold standard for the diagnosis of DLB is pathology, and there are no biochemical tests to confirm the diagnosis during the lifetime of the patient, unlike the CSF biomarkers that exist for AD. There is therefore a risk of including patients with other neurodegenerative diseases or with the association of AD and DLB. However, McKeith’s criteria have very good specificity (98 %) [76]), and the majority of our patients had a lumbar puncture, which excluded AD. Another limitation is that we did not perform specific evaluations to determine the social impact of the ToM impairments. It would be interesting in a further study to investigate this impact on daily living using questionnaires.

## Conclusions

In this study, we demonstrate ToM impairments in patients with DLB. These deficits were present at an early stage of the disease and affected both the affective and cognitive components of ToM. Impairments were correlated with atrophy in regions classically involved in ToM (prefrontal and orbitofrontal cortex, TPJ, precuneus and insula), which reinforces the hypothesis of a neuronal network of ToM. Further studies are now needed to confirm these first results and to better understand the neural correlates of ToM impairments and their functional and anatomical connectivity.

## Additional files

Additional file 1:
**Voxel-based morphometric analysis of global atrophy in the dementia with Lewy bodies group or the Alzheimer’s disease group compared with the healthy control group.** (DOCX 15 kb) Additional file 2:
**Voxel-based morphometric analysis of global atrophy in the dementia with Lewy bodies group compared with the Alzheimer’s disease group.** (DOCX 14 kb)

## Abbreviations

Aβ_42_: amyloid-β_42_
AD: Alzheimer’s disease
BA: Brodmann’s area
CFR: cued free recall
CQ: control questions
CSF: cerebrospinal fluid
CTR: cued total recall
DLB: dementia with Lewy bodies
DMS: Delayed Matching to Sample
DO: Dénomination Orale (an oral naming test)
DSST: digit symbol substitution test
FAB: Frontal Assessment Battery
FCSRT: Free and Cued Selective Reminding Test
FPR: Faux Pas Recognition
FR: free recall
HC: healthy control subjects
IADL: instrumental activities of daily living
IATI: INNOTEST Amyloid Tau Index
IR: immediate recall
MMSE: Mini Mental State Examination
MNI: Montreal Neurological Institute
MRI: magnetic resonance imaging
PD: Parkinson’s disease
RME: Reading the Mind in the Eyes
SEA: Social cognition and Emotional Assessment
SPECT: single-photon emission computed tomography
SPM: statistical parametric mapping
STS: superior temporal sulcus
TMT: Trail Making Test
ToM: theory of mind
TPJ: temporoparietal junction
TR: total recall
VBM: voxel-based morphometry
VOSP: Visual Object and Space Perception Battery

## References

[1] Shamay-Tsoory SG, Aharon-Peretz J, Perry D (2009). Two systems for empathy: a double dissociation between emotional and cognitive empathy in inferior frontal gyrus versus ventromedial prefrontal lesions. Brain..

[2] Kemp J, Despres O, Sellal F, Dufour A (2012). Theory of mind in normal ageing and neurodegenerative pathologies. Ageing Res Rev..

[3] Carrington SJ, Bailey AJ (2009). Are there theory of mind regions in the brain? A review of the neuroimaging literature. Hum Brain Mapp..

[4] Poletti M, Enrici I, Adenzato M (2012). Cognitive and affective theory of mind in neurodegenerative diseases: neuropsychological, neuroanatomical and neurochemical levels. Neurosci Biobehav Rev..

[5] Sebastian CL, Fontaine NM, Bird G, Blakemore SJ, Brito SA, McCrory EJ (2012). Neural processing associated with cognitive and affective theory of mind in adolescents and adults. Soc Cogn Affect Neurosci..

[6] Kalbe E, Schlegel M, Sack AT, Nowak DA, Dafotakis M, Bangard C (2010). Dissociating cognitive from affective theory of mind: a TMS study. Cortex..

[7] Baron-Cohen S, Ring HA, Wheelwright S, Bullmore ET, Brammer MJ, Simmons A (1999). Social intelligence in the normal and autistic brain: an fMRI study. Eur J Neurosci..

[8] Frith CD, Corcoran R (1996). Exploring ‘theory of mind’ in people with schizophrenia. Psychol Med..

[9] Gregory C, Lough S, Stone V, Erzinclioglu S, Martin L, Baron-Cohen S (2002). Theory of mind in patients with frontal variant frontotemporal dementia and Alzheimer’s disease: theoretical and practical implications. Brain..

[10] Henry JD, Phillips LH, von Hippel C (2014). A meta-analytic review of theory of mind difficulties in behavioural-variant frontotemporal dementia. Neuropsychologia..

[11] Youmans G, Bourgeois M (2010). Theory of mind in individuals with Alzheimer-type dementia. Aphasiology..

[12] Funkiewiez A, Bertoux M, de Souza LC, Levy R, Dubois B (2012). The SEA (Social Cognition and Emotional Assessment): a clinical neuropsychological tool for early diagnosis of frontal variant of frontotemporal lobar degeneration. Neuropsychology..

[13] Bodden ME, Dodel R, Kalbe E (2010). Theory of mind in Parkinson’s disease and related basal ganglia disorders: a systematic review. Mov Disord..

[14] Bodden ME, Mollenhauer B, Trenkwalder C, Cabanel N, Eggert KM, Unger MM (2010). Affective and cognitive theory of mind in patients with ’Parkinson’s disease. Parkinsonism Relat Disord..

[15] Roca M, Torralva T, Gleichgerrcht E, Chade A, Arevalo GG, Gershanik O (2010). Impairments in social cognition in early medicated and unmedicated Parkinson disease. Cogn Behav Neurol..

[16] Allain P, Havet-Thomassin V, Verny C, Gohier B, Lancelot C, Besnard J (2011). Evidence for deficits on different components of theory of mind in Huntington’s disease. Neuropsychology..

[17] Meier SL, Charleston AJ, Tippett LJ (2010). Cognitive and behavioural deficits associated with the orbitomedial prefrontal cortex in amyotrophic lateral sclerosis. Brain..

[18] Kluger BM, Heilman KM (2007). Dysfunctional facial emotional expression and comprehension in a patient with corticobasal degeneration. Neurocase..

[19] Duval C, Bejanin A, Piolino P, Laisney M, de La Sayette V, Belliard S (2012). Theory of mind impairments in patients with semantic dementia. Brain..

[20] Ghosh BC, Calder AJ, Peers PV, Lawrence AD, Acosta-Cabronero J, Pereira JM (2012). Social cognitive deficits and their neural correlates in progressive supranuclear palsy. Brain..

[21] Roca M, Manes F, Gleichgerrcht E, Ibanez A, de Toledo MEG, Marenco V (2014). Cognitive but not affective theory of mind deficits in mild relapsing-remitting multiple sclerosis. Cogn Behav Neurol..

[22] McKeith I (2004). Dementia with Lewy bodies. Dialogues Clin Neurosci..

[23] Perri R, Monaco M, Fadda L, Caltagirone C, Carlesimo GA (2014). Neuropsychological correlates of behavioral symptoms in Alzheimer’s disease, frontal variant of frontotemporal, subcortical vascular, and Lewy body dementias: a comparative study. J Alzheimers Dis..

[24] Modinos G, Obiols JE, Pousa E, Vicens J (2009). Theory of mind in different dementia profiles. J Neuropsychiatry Clin Neurosci..

[25] McKeith IG, Dickson DW, Lowe J, Emre M, O’Brien JT, Feldman H (2005). Diagnosis and management of dementia with Lewy bodies: third report of the DLB Consortium. Neurology..

[26] American Psychiatric Association (2013). Diagnostic and statistical manual of mental disorders.

[27] Dubois B, Feldman HH, Jacova C, DeKosky ST, Barberger-Gateau P, Cummings J (2007). Research criteria for the diagnosis of Alzheimer’s disease: revising the NINCDS-ADRDA criteria. Lancet Neurol..

[28] Movement Disorder Society Task Force on Rating Scales for Parkinson’s Disease (2003). The Unified Parkinson’s Disease Rating Scale (UPDRS): status and recommendations. Mov Disord.

[29] Ferman TJ, Smith GE, Boeve BF, Ivnik RJ, Petersen RC, Knopman D (2004). DLB fluctuations: specific features that reliably differentiate DLB from AD and normal aging. Neurology..

[30] Walker MP, Ayre GA, Cummings JL, Wesnes K, McKeith IG, O’Brien JT (2000). The Clinician Assessment of Fluctuation and the One Day Fluctuation Assessment Scale: two methods to assess fluctuating confusion in dementia. Br J Psychiatry..

[31] Folstein MF, Folstein SE, McHugh PR (1975). “Mini-mental state”: a practical method for grading the cognitive state of patients for the clinician. J Psychiatr Res..

[32] Grober E, Buschke H (1987). Genuine memory deficits in dementia. Dev Neuropsychol..

[33] Buschke H (1984). Cued recall in amnesia. J Clin Neuropsychol..

[34] Barbeau E, Didic M, Tramoni E, Felician O, Joubert S, Sontheimer A (2004). Evaluation of visual recognition memory in MCI patients. Neurology..

[35] Deloche G, Hannequin D. DO 80: Test de dénomination orale d’images. Paris: Les Editions du Centre de Psychologie Appliquée (ECPA); 1997.

[36] Dubois B, Slachevsky A, Litvan I, Pillon B (2000). The FAB: a Frontal Assessment Battery at bedside. Neurology..

[37] Tombaugh TN (2004). Trail Making Test A and B: normative data stratified by age and education. Arch Clin Neuropsychol..

[38] Wechsler D (2008). Wechsler Adult Intelligence Scale.

[39] Cardebat D, Doyon B, Puel M, Goulet P, Joanette Y (1990). Formal and semantic lexical evocation in normal subjects. Performance and dynamics of production as a function of sex, age and educational level. Acta Neurol Belg.

[40] Mahieux-Laurent F, Fabre C, Galbrun E, Dubrulle A, Moroni C, groupe de réflexion sur les praxies du CMRR Ile-de-France Sud. Validation of a brief screening scale evaluating praxic abilities for use in memory clinics: evaluation in 419 controls, 127 mild cognitive impairment and 320 demented patients [in French]. Rev Neurol (Paris). 2009;165:560–7. 10.1016/j.neurol.2008.11.016 19150097

[41] Rey A (1959). Manuel du Test de copie et de reproduction de mémoire de figures géométriques complexes.

[42] Rapport LJ, Millis SR, Bonello PJ (1998). Validation of the Warrington theory of visual processing and the Visual Object and Space Perception Battery. J Clin Exp Neuropsychol..

[43] Stone VE, Baron-Cohen S, Knight RT (1998). Frontal lobe contributions to theory of mind. J Cogn Neurosci..

[44] Baron-Cohen S, Wheelwright S, Hill J, Raste Y, Plumb I (2001). The “Reading the Mind in the Eyes” Test revised version: a study with normal adults, and adults with Asperger syndrome or high-functioning autism. J Child Psychol Psychiatry..

[45] Ekman P, Friesen W (1975). Pictures of facial affect.

[46] Bertoux M, Volle E, Funkiewiez A, de Souza LC, Leclercq D, Dubois B (2012). Social Cognition and Emotional Assessment (SEA) is a marker of medial and orbital frontal functions: a voxel-based morphometry study in behavioral variant of frontotemporal degeneration. J Int Neuropsychol Soc..

[47] Ruffman T, Henry JD, Livingstone V, Phillips LH (2008). A meta-analytic review of emotion recognition and aging: implications for neuropsychological models of aging. Neurosci Biobehav Rev..

[48] Shamay-Tsoory SG, Tomer R, Berger BD, Goldsher D, Aharon-Peretz J (2005). Impaired “affective theory of mind” is associated with right ventromedial prefrontal damage. Cogn Behav Neurol..

[49] Shamay-Tsoory SG, Aharon-Peretz J (2007). Dissociable prefrontal networks for cognitive and affective theory of mind: a lesion study. Neuropsychologia..

[50] Cuerva AG, Sabe L, Kuzis G, Tiberti C, Dorrego F, Starkstein SE (2001). Theory of mind and pragmatic abilities in dementia. Neuropsychiatry Neuropsychol Behav Neurol..

[51] Zaitchik D, Koff E, Brownell H, Winner E, Albert M (2006). Inference of beliefs and emotions in patients with Alzheimer’s disease. Neuropsychology..

[52] Fernandez-Duque D, Baird JA, Black SE (2009). False-belief understanding in frontotemporal dementia and Alzheimer’s disease. J Clin Exp Neuropsychol..

[53] Castelli I, Pini A, Alberoni M, Liverta-Sempio O, Baglio F, Massaro D (2011). Mapping levels of theory of mind in Alzheimer’s disease: a preliminary study. Aging Ment Health..

[54] Laisney M, Bon L, Guiziou C, Daluzeau N, Eustache F, Desgranges B (2013). Cognitive and affective theory of mind in mild to moderate Alzheimer’s disease. J Neuropsychol..

[55] Narme P, Mouras H, Roussel M, Devendeville A, Godefroy O (2013). Assessment of socioemotional processes facilitates the distinction between frontotemporal lobar degeneration and Alzheimer’s disease. J Clin Exp Neuropsychol..

[56] Aboulafia-Brakha T, Christe B, Martory MD, Annoni JM (2011). Theory of mind tasks and executive functions: a systematic review of group studies in neurology. J Neuropsychol..

[57] Beyer MK, Larsen JP, Aarsland D (2007). Gray matter atrophy in Parkinson disease with dementia and dementia with Lewy bodies. Neurology..

[58] Whitwell JL, Weigand SD, Shiung MM, Boeve BF, Ferman TJ, Smith GE (2007). Focal atrophy in dementia with Lewy bodies on MRI: a distinct pattern from Alzheimer’s disease. Brain..

[59] Zhong J, Pan P, Dai Z, Shi H (2014). Voxelwise meta-analysis of gray matter abnormalities in dementia with Lewy bodies. Eur J Radiol..

[60] Stuss DT, Levine B (2002). Adult clinical neuropsychology: lessons from studies of the frontal lobes. Annu Rev Psychol..

[61] Platek SM, Keenan JP, Gallup GG, Mohamed FB (2004). Where am I? The neurological correlates of self and other. Brain Res Cogn Brain Res..

[62] Adenzato M, Cavallo M, Enrici I (2010). Theory of mind ability in the behavioural variant of frontotemporal dementia: an analysis of the neural, cognitive, and social levels. Neuropsychologia..

[63] Baglio F, Castelli I, Alberoni M, Blasi V, Griffanti L, Falini A (2012). Theory of mind in amnestic mild cognitive impairment: an fMRI study. J Alzheimers Dis..

[64] Hynes CA, Baird AA, Grafton ST (2006). Differential role of the orbital frontal lobe in emotional versus cognitive perspective-taking. Neuropsychologia..

[65] Shamay-Tsoory SG, Harari H, Aharon-Peretz J, Levkovitz Y (2010). The role of the orbitofrontal cortex in affective theory of mind deficits in criminal offenders with psychopathic tendencies. Cortex..

[66] Bodden ME, Kubler D, Knake S, Menzler K, Heverhagen JT, Sommer J (2013). Comparing the neural correlates of affective and cognitive theory of mind using fMRI: Involvement of the basal ganglia in affective theory of mind. Adv Cogn Psychol..

[67] Geraci A, Surian L, Ferraro M, Cantagallo A (2010). Theory of mind in patients with ventromedial or dorsolateral prefrontal lesions following traumatic brain injury. Brain Inj..

[68] Lee TM, Ip AK, Wang K, Xi CH, Hu PP, Mak HK (2010). Faux pas deficits in people with medial frontal lesions as related to impaired understanding of a speaker’s mental state. Neuropsychologia..

[69] Kalbe E, Grabenhorst F, Brand M, Kessler J, Hilker R, Markowitsch HJ (2007). Elevated emotional reactivity in affective but not cognitive components of theory of mind: a psychophysiological study. J Neuropsychol..

[70] Schurz M, Radua J, Aichhorn M, Richlan F, Perner J (2014). Fractionating theory of mind: a meta-analysis of functional brain imaging studies. Neurosci Biobehav Rev..

[71] Uddin LQ, Molnar-Szakacs I, Zaidel E, Iacoboni M (2006). rTMS to the right inferior parietal lobule disrupts self-other discrimination. Soc Cogn Affect Neurosci..

[72] Decety J, Lamm C (2007). The role of the right temporoparietal junction in social interaction: how low-level computational processes contribute to meta-cognition. Neuroscientist..

[73] Cavanna AE, Trimble MR (2006). The precuneus: a review of its functional anatomy and behavioural correlates. Brain..

[74] Fusar-Poli P, Placentino A, Carletti F, Landi P, Allen P, Surguladze S (2009). Functional atlas of emotional faces processing: a voxel-based meta-analysis of 105 functional magnetic resonance imaging studies. J Psychiatry Neurosci..

[75] Gallagher HL, Happe F, Brunswick N, Fletcher PC, Frith U, Frith CD (2000). Reading the mind in cartoons and stories: an fMRI study of ‘theory of mind’ in verbal and nonverbal tasks. Neuropsychologia..

[76] Nelson PT, Jicha GA, Kryscio RJ, Abner EL, Schmitt FA, Cooper G (2010). Low sensitivity in clinical diagnoses of dementia with Lewy bodies. J Neurol..

