# Supplementary material for: Cognitive and affective theory of mind in dementia with Lewy bodies and Alzheimer’s disease
Source: Alzheimers Res Ther. 2016 Mar 16;8:10. doi: 10.1186/s13195-016-0179-9 (PMC4793654; doi:10.1186/s13195-016-0179-9)
Supplement: Additional file 1: — Voxel-based morphometric analysis of global atrophy in the dementia with Lewy bodies group or the Alzheimer’s disease group compared with the healthy control group. (DOCX 15 kb) [file 13195_2016_179_MOESM1_ESM.docx]

**Additional file 1**

**Voxel-based morphometry analysis of global atrophy in the dementia with Lewy bodies group or the Alzheimer’s disease group compared to the healthy control group**

| **Anatomical region** | | | **BA** | **R/L** | **Coordinates** | | | **T-value** | **Cluster size**  ***P*<0.001, uncorr** | **Cluster size**  ***P*<0.05,**  **FWE correc** |
| --- | --- | --- | --- | --- | --- | --- | --- | --- | --- | --- |
|  |  |  |  |  | **X** | **Y** | **Z** |  |  |  |
| **DLB group vs. HC group** | | |  |  |  |  |  |  |  |  |
|  | **Frontal lobe** | |  |  |  |  |  |  |  |  |
|  | | Med. front. gyrus / ACC | 10/32 | R | 8 | 56 | 9 | 6.01 | 2494 | / |
|  | | Mid. front. gyrus | 9 | R | 42 | 26 | 40 | 5.79 | 90 | / |
|  | | " | 9 | L | -45 | 20 | 97 | 5.65 | 519 | / |
|  | | " | 11 | R | 36 | 47 | -12 | 5.58 | 231 | / |
|  | | " | 46 | L | -42 | 33 | 18 | 4.60 | 89 | / |
|  | | Inf. front. gyrus | 10 | L | -40 | 44 | 1 | 4.70 | 224 | / |
|  | | Inf. front. gyrus / insula | 47/13 | R | 32 | 21 | -14 | 6.29 | 1464 | / |
|  | | " | 47/13 | L | -27 | 20 | -18 | 5.16 | 594 | / |
|  | | Orbital gyrus | 11 | R | 6 | 48 | -24 | 5.79 | 279 | / |
|  | **Temporal lobe** | |  |  |  |  |  |  |  |  |
|  | | Sup. temp. gyrus | 38 | L | -56 | 2 | -12 | 6.08 | 676 | / |
|  | | " | 22 | R | 62 | -39 | 18 | 4.52 | 52 | / |
|  | | Inf. temp. gyrus | 20 | L | -42 | -1 | -42 | 5.01 | 270 | / |
| **AD group vs HC group** | | |  |  |  |  |  |  |  |  |
|  | **Frontal lobe** | |  |  |  |  |  |  |  |  |
|  | | Sup. front. gyrus | 10 | L | -24 | 50 | 28 | 6.16 | 142 | / |
|  | | " | 6 | L | -24 | 8 | 55 | 5.96 | 96 | / |
|  | | " | 9 | R | 12 | 53 | 40 | 5.81 | 91 | / |
|  | | Mid. front. gyrus | 8 | R | -28 | 29 | 46 | 6.81 | 426 | / |
|  | | Med. front. gyrus | 32 | L | -6 | 6 | 52 | 6.15 | 71 | / |
|  | | Cingulate gyrus | 31 | R | 9 | -45 | 42 | 5.83 | 401 | / |
|  | **Temporal lobe** | |  |  |  |  |  |  |  |  |
|  | | Sup. temp. gyrus | 22 | R | 58 | -3 | 1 | 5.87 | 294 | / |
|  | | " | 22 | R | 49 | -40 | 13 | 5.61 | 140 | / |
|  | | Fusiform gyrus | 19 | R | 26 | -69 | -12 | 6.90 | 127 | / |
|  | | Fusiform gyrus / Parahippocampal / Hippocampus | 28 | L | -26 | -13 | -35 | 11.11 | 9117 | 85 |
|  | | " | 28 | R | 26 | -10 | -36 | 9.22 | 5809 | 67 |
|  | **Insula** | | 13 | R | 39 | 11 | -0 | 5.57 | 602 | / |

ACC = anterior cingulate cortex; AD = Alzheimer’s disease; BA = Brodmann’s area; DLB = dementia with Lewy bodies; Front. = frontal; HC = healthy controls; Inf. = inferior; Med. = medial; Mid. = middle; R/L = Right/Left; Sup. = superior; Temp. = temporal.
