# Supplementary material for: Cognitive and affective theory of mind in dementia with Lewy bodies and Alzheimer’s disease
Source: Alzheimers Res Ther. 2016 Mar 16;8:10. doi: 10.1186/s13195-016-0179-9 (PMC4793654; doi:10.1186/s13195-016-0179-9)
Supplement: Additional file 2: — Voxel-based morphometric analysis of global atrophy in the dementia with Lewy bodies group compared with the Alzheimer’s disease group. (DOCX 14 kb) [file 13195_2016_179_MOESM2_ESM.docx]

**Additional file 2**

**Voxel-based morphometry analysis of global atrophy in the dementia with Lewy bodies group compared to the Alzheimer’s disease group**

| **Anatomical region** | | **BA** | **R/L** | **Coordinates** | | | **T-value** | **Cluster size** |
| --- | --- | --- | --- | --- | --- | --- | --- | --- |
|  |  |  |  | **X** | **Y** | **Z** |  |  |
| **Atrophy in the AD group compared to the DLB group** | | | | | | | | |
| **Frontal lobe** | |  |  |  |  |  |  |  |
|  | Mid. Front. gyrus | 8 | L | -34 | 27 | 45 | 4.94 | 68 |
|  | " | 6 | L | -26 | 6 | 57 | 4.90 | 74 |
| **Parietal lobe** | |  |  |  |  |  |  |  |
|  | Sup. parietal lobule | 7 | R | 33 | -49 | 49 | 4.82 | 55 |
|  | Inf. parietal lobule | 40 | L | -46 | -42 | 46 | 4.97 | 117 |
|  | Precuneus | 7 | L | -3 | -61 | 45 | 4.65 | 105 |
| **Occipital lobe** | |  |  |  |  |  |  |  |
|  | Cuneus | 18 | R | 22 | -90 | 22 | 5.35 | 229 |
|  | Lingual gyrus | 18 | L | -27 | -75 | -15 | 5.17 | 147 |
| **Temporal lobe** | |  |  |  |  |  |  |  |
|  | Supramarginal gyrus | 40 | R | 54 | -54 | 22 | 4.75 | 176 |
|  | Mid. temp. gyrus | 39 | L | -56 | -58 | 10 | 4.63 | 158 |
|  | Fusiform gyrus | 19 | R | 26 | -69 | -12 | 6.33 | 1589 |
|  | " | 28 | L | -26 | -10 | -38 | 4.78 | 185 |
|  | Amygdala / Hippocampus |  | R | 24 | -10 | -11 | 5.69 | 664 |
|  | Hippocampus / Parahippocampal | 28 | L | -21 | -10 | -14 | 5.32 | 611 |
|  | Parahippocampal | 35 | L | -27 | -28 | -27 | 4.49 | 129 |
| **Atrophy in the DLB group compared to the AD group** | | | | | | | | |
|  | Cingulate gyrus | 24 | L | -9 | 5 | 28 | 5.19 | 601 |
|  | Middle frontal gyrus | 8 | R | 52 | 15 | 43 | 4.97 | 112 |

AD: Alzheimer’s disease, BA: Brodmann’s area, DLB: dementia with Lewy bodies, Front.: frontal, Inf.: inferior, Mid: middle, R/L: Right/Left, Sup.: superior, Temp.: temporal
